# Annotations

**Published:** 1892-05-28

**Authors:** 


### ANNOTATIONS.

**Trades Unionism Run Mad.**

A SHORT time ago a two days' demonstration was given in Canning Town on behalf of the funds of the Albert Dock Hospital, a branch of the Seamen's Hospital at Greenwich, which was established on the opposite side of the water for the greater convenience of the sailors and other workmen in that neighbourhood. The demonstration was heartily supported by several friendly and trade societies, and was highly popular in the neighbourhood, and decidedly successful. But it will hardly be believed that two or three trades unions protested against the proceedings, and ostentatiously avowed their determination to have nothing to do with them, and gave as their only reason the fact that non-union men and their wives and children are treated at the hospital exactly as if they were good trades unionists. Now it is too late in the day for any instructed person to speak against trades unions. They are necessary and useful institutions when conducted with sense and manliness. One would not even think it worth while to notice the fact if two or three isolated individuals had been so foolish as to try to exclude poor men and their wives and children from hospital benefits because they did not belong to trade societies. But what shall be said of whole unions, with their leaders, in their corporate and official capacity, acting after this fashion? One cannot believe that the leaders are Englishmen: they themselves would probably scorn to be called Christians. Mere words would have no effect upon stupid and vicious persons of this strange character. One cannot but see, however, in facts of this kind the danger that lurks in the massing together of tens of thousands of workpeople without the admixture of better educated and brighter-minded classes. There is a sort of moral insanity in such a sullen and destructive temper; there is murder in it. The workman who gives way to it is morally as bad as a savage beast, and as dangerous. The policeman is the only kind of civilised institution he can understand. Fair fighting, English manliness, live and let live, are things his dull intellect is not capable of comprehending. As for Christian charity, it would be "like a jewel of gold in a swine's snout" in the possession of such a boor. One is ashamed to think that such dull-wittedness and senseless inhumanity can be found in Englishmen.

**An Extraordinary Lancashire Man.**

DR. WILKINSON, Junior House Surgeon at the Bolton Infirmary, was sued last week in the Bolton County Court for £10, the alleged value of an amputated arm. The case, so far as we know, was new, and it has a decidedly Lancashire ring about it. Your genuine north-countryman loves money, and will sometimes take very peculiar opportunities of trying to get it. It seems that a boy named Housley, having been injured in an accident, had been taken to the infirmary, and there one of his arms had been amputated. What was done with the arm it is not stated; but the father of the boy asked that it might be given to him, and made a point of having his request complied with. No reason is forthcoming why the house surgeon declined to give up the limb; but we can very well understand that there might be reasons of good and sufficient character. As bearing on the point it is desirable to remember what is the practically universal custom in such cases. The custom is for the hospital authorities to be responsible for the decent disposal of amputated limbs: and in the absence of statutory or other authoritative guidance, it would seem that custom should regulate the law. The father of the boy did not, however, take this view of the question, but demanded that the amputated arm should be given up to him. When his request was not complied with, he made the claim, already indicated, for £10 from Dr. Wilkinson as the value which he chose to put upon it. Such a claim was altogether ridiculous. It seems, however, that the father had already recovered £40 under Lord Campbell's Act for the death of his son; and there was an evident disposition on his part to make as much money out of the whole business as possible. The judge decided against the claim, and Dr. Wilkinson was accorded a verdict with costs. But what kind of a father can he be who acts after this fashion towards a doctor and a charitable institution that have provided his injured son with a home, given him the best resources of medical and surgical science free of cost, and tenderly nursed and cared for him to the last day of his life? One really does not know what to make of such a man. Of course, his intelligence must be of the feeblest; but even feeble-minded people ought to have some sense of gratitude, some feeling of obligation. The very dog is considered outrageously vicious that snaps at the hand of its benefactor. But this extraordinary Lancashire man seems to have been absolutely and entirely destitute of gratitude, sense, and shame. Very few people would think it desirable to subscribe to hospitals if there are many fathers of the type of Mr. Housley.

**Another Cancer Cure.**

DR. SCHULTZ, of Buda-Pesth, has made some experiments with alcohol in the treatment of cancer. The alcohol is injected into the structure of the cancerous growth. A certain amount of benefit seems to have been gained. About ten cases have been treated in all; and although it is not claimed that any positive cures have been effected, it is asserted that in several cases the further growth of the cancerous structure has been arrested. This, as will be seen, does not really amount to very much. In certain cases months may pass without the observer being able to say with certainty whether a particular tumour is growing larger or not. That is especially the case when the growth is internal; and all the cases treated by Dr. Schultz appear to have been of this character. So far as the experiments have gone it cannot be considered that any results of a permanently trustworthy kind have been obtained. It is satisfactory, however, to record that capable medical men in various parts of the world are constantly and conscientiously working at the subject of cancer. It is desirable also that other medical men who have opportunities should experiment on similar lines. The failure of so many methods of cancer treatment is thought by some to be somewhat of a blot on the medical escutcheon. Experienced physicians and surgeons can hardly endorse that view of the question. Nevertheless, we are all thankful when new lines are struck out, and when there is any promise, however faint, of the dawn of a brighter day for cancer patients. It is not impossible that the real hope of the future, so far as dangerous diseases are concerned, lies rather in certain fundamental changes that require to be made in the rearing, and even in the breeding of the human race. In going forward civilisation must go backward in some respects if it is to make a real advance.

